# Community Structure and Abundance of Archaea in a *Zostera marina* Meadow: A Comparison between Seagrass-Colonized and Bare Sediment Sites

**DOI:** 10.1155/2019/5108012

**Published:** 2019-11-18

**Authors:** Pengfei Zheng, Chuantao Wang, Xiaoli Zhang, Jun Gong

**Affiliations:** ^1^CAS Key Laboratory of Coastal Environmental Processes and Ecological Remediation, Yantai Institute of Coastal Zone Research, Chinese Academy of Sciences, Yantai 264003, China; ^2^University of Chinese Academy of Sciences, Beijing 100049, China; ^3^Shandong Oriental Ocean Sci-Tech Co., Ltd., Yantai 264003, China; ^4^Laboratory of Microbial Ecology and Matter Cycles, School of Marine Sciences, Sun Yat-Sen University, Zhuhai 519082, China; ^5^Southern Marine Science and Engineering Guangdong Laboratory (Zhuhai), Zhuhai 519000, China

## Abstract

Seagrass colonization alters sediment physicochemical properties by depositing seagrass fibers and releasing organic carbon and oxygen from the roots. How this seagrass colonization-induced spatial heterogeneity affects archaeal community structure and abundance remains unclear. In this study, we investigated archaeal abundance, diversity, and composition in both vegetated and adjacent bare surface sediments of a *Zostera marina* meadow. High-throughput sequencing of 16S rDNA showed that *Woesearchaeota*, *Bathyarchaeota*, and *Thaumarchaeota* were the most abundant phyla across all samples, accounting for approximately 42%, 21%, and 17% of the total archaeal communities, respectively. In terms of relative abundance, *Woesearchaeota* and *Bathyarchaeota* were not significantly different between these two niches; however, specific subclades (Woese-3, Woese-21, Bathy-6, Bathy-18) were significantly enriched in vegetated sediments (*P* < 0.05), while *Thaumarchaeota* was favored in unvegetated sites (*P* = 0.02). The quantification of archaeal 16S rRNA genes showed that the absolute abundance of the whole archaeal community, *Bathyarchaeota*, and Woese-3, Woese-10, Woese-13, and Woese-21 was significantly more abundant in vegetated sediments than in bare sediments (*P* < 0.05). Our study expands the available knowledge of the distribution patterns and niche preferences of archaea in seagrass systems, especially for the different subclades of *Woesearchaeota* and *Bathyarchaeota*, in terms of both relative proportions and absolute quantities.

## 1. Introduction

Seagrass meadows support high primary productivity, playing an essential role in shaping coastal ecology [1]. The ecological importance of seagrass meadows is well recognized because of their burial and sequestration of organic carbon in sediments, which contributes to mitigating atmospheric CO_2_ increases [2, 3]. Seagrass meadows also trap organic particles from seawater and exude labile dissolved organic carbon (DOC) through seagrass roots, giving rise to an organic matter-rich rhizosphere [4]. Furthermore, during photosynthesis, the roots release a portion of O_2_ to sediments, which creates a microzone of elevated oxygen concentrations in rhizosphere sediments compared with surrounding unvegetated sediments [5, 6].

These geochemical characteristics of the seagrass rhizosphere may significantly affect the spatial distribution and ecological function of both bacteria and archaea [7]. For example, a greater abundance of the total bacterial community, increased sulfate-reducing activities [8, 9], and higher diversity and abundance of specific bacterial lineages (e.g., diazotrophs) [10] were usually detected in vegetated sediments compared with unvegetated sediments, though the overall bacterial community structure was not significantly different between these two niches [10]. Cifuentes et al. [11] investigated benthic archaeal diversity in a *Zostera noltii* meadow using clone library and sequencing and found that *Methanobacteria* dominated the community. However, little is known about archaeal diversity and spatial distributions in seagrass ecosystems.

*Woesearchaeota* (formerly known as DHVEG-6, [12]) and *Bathyarchaeota* (formerly MCG, [13]) are two common archaeal groups detected in various organic matter-rich sediments with high proportions, such as estuaries [14], seafloors [15], and mangrove sediments [16]. To thoroughly understand their ecological distribution and significance, the two groups were further divided into subclades based on phylogenetic analyses of their 16S rRNA genes.

Recently, phylogenetic analysis has shown that the phyla *Woesearchaeota* and *Bathyarchaeota* include 26 and 25 subclades, designated as Woese-1 to Woese-26 and Bathy-1 to Bathy-25, respectively [12, 13]. On a worldwide scale, these subclades exhibit distinct habitat characteristics (e.g., anoxic/oxic, marine/freshwater, sediment depth layers) [12, 13, 17, 18]. In a specific environment, some specific environmental factors regulate their distributions. For example, in mangrove wetlands, pH was found to be the major factor shaping the Bathyarchaeotal community structure, and Bathy-6 preferentially occurred in slightly acidic and high (total organic carbon) TOC sediments [16]. In the White Oak River estuary, Bathy-6 was found to mainly persist in sulfide-depleted shallow sediments [14]. However, the niche preference of various subclades of *Woesearchaeota* and *Bathyarchaeota* in seagrass meadows is unknown [16, 19–21].

In this study, we hypothesized that distinct archaeal abundances and community structures occur in seagrass-vegetated and adjacent bare sediments. To verify the hypothesis, surface (0-5 cm) sediment samples were collected from a temperate seagrass (*Zostera marina*) meadow, and archaeal community diversity, composition, and abundance were compared between the two niches through high-throughput sequencing and qPCR. Additionally, the spatial heterogeneity of the seagrass meadow provides a unique opportunity to explore the distribution patterns of different subclades recently recognized in *Woesearchaeota* and *Bathyarchaeota* in terms of both relative proportions and absolute quantities.

## 2. Materials and Methods

### 2.1. Study Area, Sampling, and Determination of Environmental Parameters

The study area (37°21′1.46^″^ N, 122°34′26.96^″^ E), sampling processes, and measurements of physiochemical parameters in the *Z. marina* seagrass meadow were as previously described [10]. Briefly, three (V1-V3) surface (0-5 cm) sediment samples were randomly collected from the seagrass-vegetated region, and another three control (U1-U3) samples were collected from the adjacent bare (unvegetated) region in the Swan Lake lagoon (Rongcheng Bay, Yellow Sea, China) in May 2013. All samples were homogenized and stored at -80°C until DNA extraction. In the vegetated sites, the overlying water had significantly higher chlorophyll *a* (Chl-*a*) contents, and the sediments had higher concentrations of metals, such as Pb, Cr, Fe, Co, Ni, Cu, and As; finer sediment grains; lower ratios of the total organic carbon (TOC) to total nitrogen (TN); and lower concentrations of ammonium (NH_4_^+^) and dissolved inorganic nitrogen (DIN) in the pore water, compared with the unvegetated sites.

### 2.2. DNA Extraction and High-Throughput Sequencing

DNA was extracted from 0.5 to 1.0 g of sediment using a FastDNA Kit for Soil (MP Biomedical, USA) according to the manufacturer's instructions. DNA integrity was checked in a 1.0% agarose gel, and the concentration was measured using a ND-2000C spectrophotometer (NanoDrop, USA).

High-throughput sequencing of archaeal 16S rRNA genes was performed to reveal archaeal diversity and the community composition in the sediments. The V3 region of the archaeal 16S rRNA gene was PCR amplified with adapter-modified core primers, which contained unique 12 bp bar codes and the archaeal-specific primers A344F (5′-GGGGYGCASCAGGSG-3′) and A519R (5′-GGTDTTACCGCGGCKGCTG-3′). PCR was conducted using the following program: 94°C for 5 min; 25 cycles of 94°C for 50 s, 53°C for 50 s, and 72°C for 50 s; and a final extension at 72°C for 6 min [22]. The amplicons were gel purified and further purified with AMPure beads (Beckman Coulter, USA) and then pooled in equimolar proportions and sequenced on 318 chips with an Ion Torrent Personal Genome Machine (PGM) according to the manufacturer's instructions (Life Technologies, USA).

### 2.3. Analysis of High-Throughput Sequencing Data

The Ion Torrent fastq files were processed via the QIIME v.1.9.0 work flow [23]. The raw reads were sorted to the corresponding samples according to the barcodes and filtered to remove reads that (i) were shorter than 110 bases, (ii) exhibited quality scores less than 20, (iii) exhibited ambiguous bases, or (iv) exhibited homopolymer runs with 6 or more bases. Both the forward and reverse primers were removed along with the barcodes. Based on the Silva database (v.128) [24], chimeras were identified using the script identify_chimeric_seqs.py. Representative operational taxonomic units (OTUs) were chosen according to a minimum sequence identity of 97% with the UCLUST program [25], and their sequences were aligned against those in the Silva database by using the PyNAST program [23]. Taxonomy was assigned at a sequence similarity of 0.97. The reads assigned to bacteria, unassigned, or singletons (the OTUs containing a single read across all samples) were discarded prior to building the OTU table. To evaluate alpha diversity estimators, we rarefied the high-quality sequences at the lowest number for all samples. The alpha diversity indexes (OTU richness, Shannon, Simpson, and Chao1) were calculated after resampling using the script alpha_diversity.py. Beta diversity was calculated based on Bray-Curtis dissimilarities and visualized using nonmetric multidimensional scaling (NMDS) in PRIMER v.6 (Primer-E, UK).

### 2.4. Phylogenetic Analysis of *Woesearchaeota* and *Bathyarchaeota* Sequences

To explore the phylogenetic relationships of all *Woesearchaeota* and *Bathyarchaeota* sequences with the subclades classified by Liu et al. [12] and Zhou et al. [13], reference sequences were downloaded from GenBank and aligned with our sequences using the MAFFT program. Maximum likelihood (ML) trees were built in the “FastTree” program with the GTRGAMMAI model, and a bootstrap analysis of 1,000 replications was applied in all phylogenetic analyses.

### 2.5. Quantitative Real-Time PCR (qPCR)

All qPCR assays were based on the fluorescence intensity of the SYBR green dye and were performed to quantify archaeal 16S rRNA gene copy numbers in the sediments as previously described [10]. qPCR was performed using the primers A931F (5′-AGGAATTGGCGGGGGAGCA-3′) and M1100R (5′-BGGGTCTCGCTCGTTRCC-3′) [26, 27], with the following program: 7 min of initial denaturation at 95°C, followed by 40 cycles of 95°C for 30 s, 64°C for 30 s, and 72°C for 30 s. The data were retrieved at 72°C, and all of the reactions were completed with a melting curve from 60°C to 95°C with increases of 0.5°C each cycle. PCR amplification was carried out in an ABI 7500 Fast Real-Time PCR System (Applied Biosystems, USA). The average PCR efficiency (*E*) for amplifying the 16S rRNA genes was 84.3%, and the correlation coefficients (*R*^2^) for all of the assays were greater than 0.90. Controls without templates resulted in undetectable values.

### 2.6. Statistical Analysis

Student's (two-tailed) *t*-tests were performed to compare the relative proportions, absolute quantities, and alpha diversities of archaea between seagrass-vegetated and unvegetated sediments using SPSS (v. 20.0) software for Windows (SPSS, Chicago, IL, USA). To assess the variances in the compositions of archaeal communities in all of the samples, nonmetric multidimensional scaling (NMDS) was conducted on the basis of a Bray-Curtis similarity matrix using the PRIMER (v.6) software package (Primer-E, United Kingdom), and the analysis of similarity (ANOSIM) was performed to statistically test the difference in archaeal community structure between vegetated and unvegetated samples.

### 2.7. Accession Numbers

The Ion Torrent PGM sequencing data of archaeal 16S rRNA genes have been deposited in the NCBI Sequence Read Archive under accession number PRJNA385281.

## 3. Results

### 3.1. Community Structure and Distribution of Archaea in the *Z. marina* Meadow

**Figure 1:**
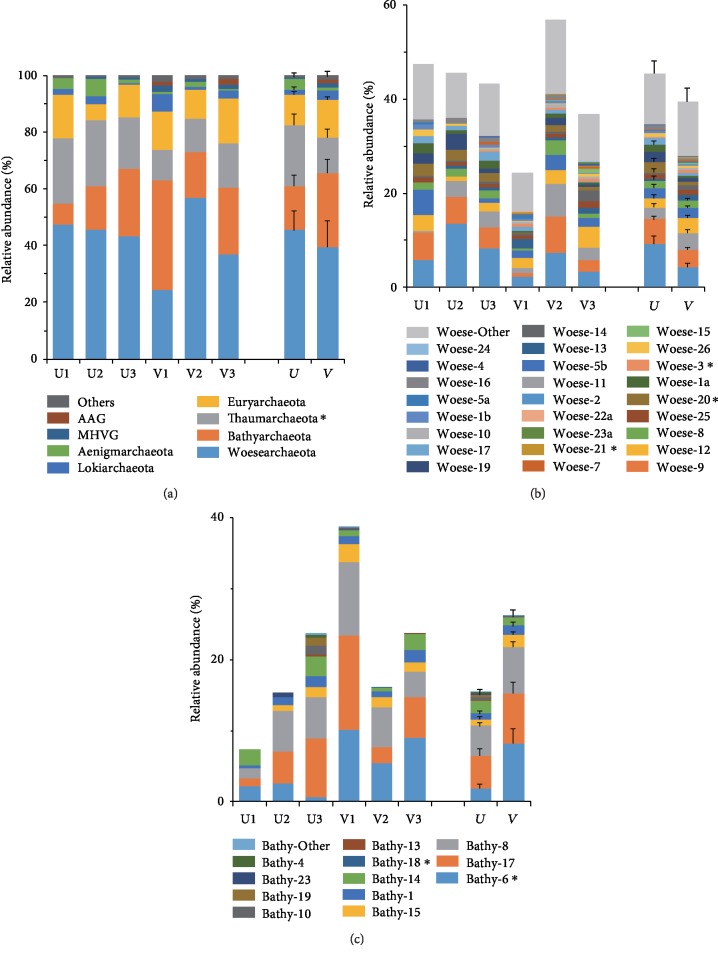
Comparison of the archaeal community composition between vegetated and unvegetated sediments: (a) at the phylum level (MHVG, Marine Hydrothermal Vent Group; AAG, Ancient Archaeal Group; Others, archaeal phyla with relative abundance < 1%); (b) subclades of *Woesearchaeota*; (c) subclades of *Bathyarchaeota*. Those taxa showing significant differences between the two niches at the 0.05 level are indicated with ^∗^.

A total of 100,636 raw reads were obtained from the 6 samples, and 33,922 reads were finally retained after quality filtering and removing chimeras and singletons ([Supplementary-material supplementary-material-1]). At a cut-off of 97% sequence similarity, a total of 4,898 OTUs were obtained, representing 13 phyla in domain Archaea. Overall, the most abundant phylum was *Woesearchaeota* (mean ± SE, 42.4% ± 4.09%; *n* = 6), followed by *Bathyarchaeota* (20.8% ± 3.98%), *Thaumarchaeota* (17.0% ± 2.03%), and *Euryarchaeota* (12.1% ± 1.42%). The two phyla *Aenigmarchaeota* (2.31% ± 0.84%) and *Lokiarchaeota* (2.59% ± 0.74%) appeared to be minor components. The remaining taxa, such as Miscellaneous Euryarchaeotic Group (MEG), WSA2, *Diapherotrites*, *Altiarchaeales*, and AK8, were rare (<1%) across all samples (Figure 1(a)).

Within *Woesearchaeota*, the sequences were clustered into 26 subclades according to the classification proposed by Liu et al. [12] ([Supplementary-material supplementary-material-1]). Woese-2 (6.79% ± 1.51%), Woese-9 (4.49% ± 0.93%), and Woese-11 (2.94% ± 0.86%) were the major subclades among all samples (Figure 1(b), [Supplementary-material supplementary-material-1]). Among *Bathyarchaeota*, 12 defined subclades [13] were detected ([Supplementary-material supplementary-material-1]), with Bathy-17 (5.87% ± 1.65%), Bathy-8 (5.39% ± 1.09%), and Bathy-6 (4.97% ± 1.44%) representing the major subclades (Figure 1(c), [Supplementary-material supplementary-material-1]). Other groups at the class level, such as Group C3 (7.63% ± 0.65%), Marine Group I (4.51% ± 1.81%), Soil Crenarchaeotic Group (SCG) (4.82% ± 1.64%), *Methanobacteria* (2.49% ± 0.95%), and *Thermoplasmata* (8.94% ± 1.41%), were much less abundant in the seagrass system ([Supplementary-material supplementary-material-1]).

Student's *t*-test results showed no significant difference in the relative proportions of most archaeal phyla except for *Thaumarchaeota*, which presented significantly higher proportions in unvegetated sediments (vegetated vs. unvegetated, 12.6% ± 1.23% vs. 21.46% ± 1.35%, *P* = 0.02) (Figure 1(a), [Supplementary-material supplementary-material-1]). Although *Woesearchaeota* showed similar proportions in the vegetated and unvegetated sediments (Figure 1(a)), its subclades Woese-3 (*P* = 0.02) and Woese-21 (*P* < 0.01) presented significantly higher proportions in the vegetated sediments, while Woese-20 showed the opposite trend (*P* = 0.02) (Figure 1(b)). The relative proportion of *Bathyarchaeota* was almost twice as high in vegetated sediments as in unvegetated sediments (Figure 1(a)); in particular, the subclades Bathy-6 and Bathy-18 were significantly enriched in vegetated sediments (*P* < 0.05, Figure 1(c)).

**Figure 2:**
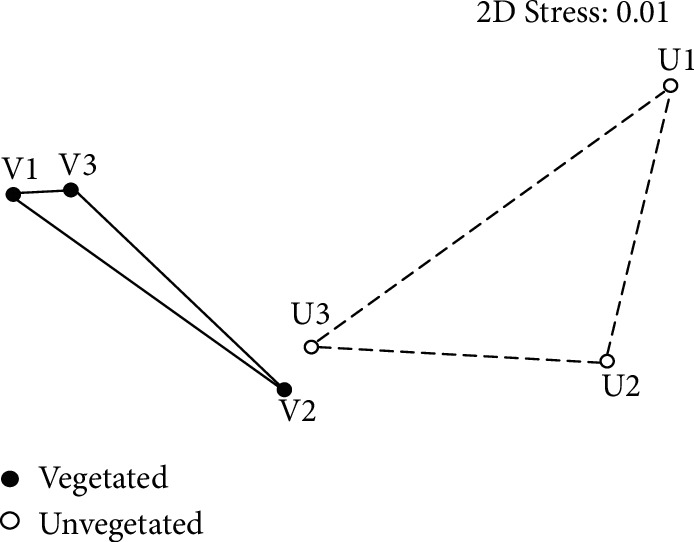
Nonmetric multidimensional scaling plots based on Bray-Curtis distance showing the differences in benthic archaeal community structure between vegetated and unvegetated sediments.

The plot of NMDS ordination showed that the vegetated samples were separated from the bare sediment samples (Figure 2). However, the difference in the overall archaeal community structure between these two types of sediments was not significant (ANOSIM, *P* = 0.10).

### 3.2. Absolute Abundance of Archaea in Seagrass-Vegetated and Unvegetated Samples

**Figure 3:**
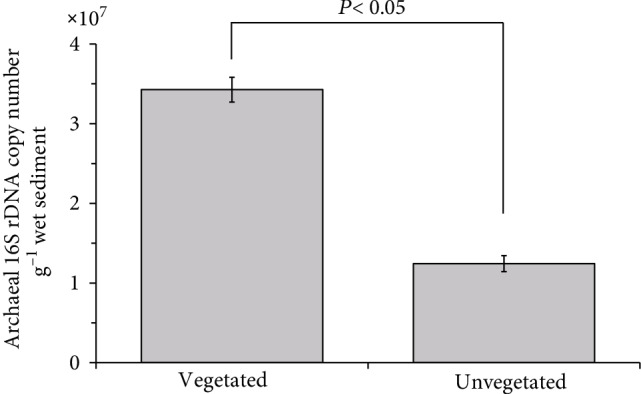
The copy numbers of archaeal 16S rRNA genes in vegetated sediments (3.42 × 10^7^ copies g^−1^ sediment on average) were significantly higher than those in unvegetated sediments (1.24 × 10^7^ copies g^−1^ sediment on average) (*P* < 0.05, *n* = 3).

The total archaeal 16S rRNA gene copy numbers varied widely across all samples, ranging from 7.6 × 10^5^ to 4.7 × 10^7^ copies g^−1^ wet sediment. The copy number of archaeal 16S rRNA gene in the vegetated sediments was (3.42 ± 0.15) × 10^7^ copies g^−1^ sediment, which was nearly three times higher than those in bare sediments ((1.24 ± 0.11) × 10^7^ copies g^−1^, *P* < 0.05, Figure 3).

**Figure 4:**
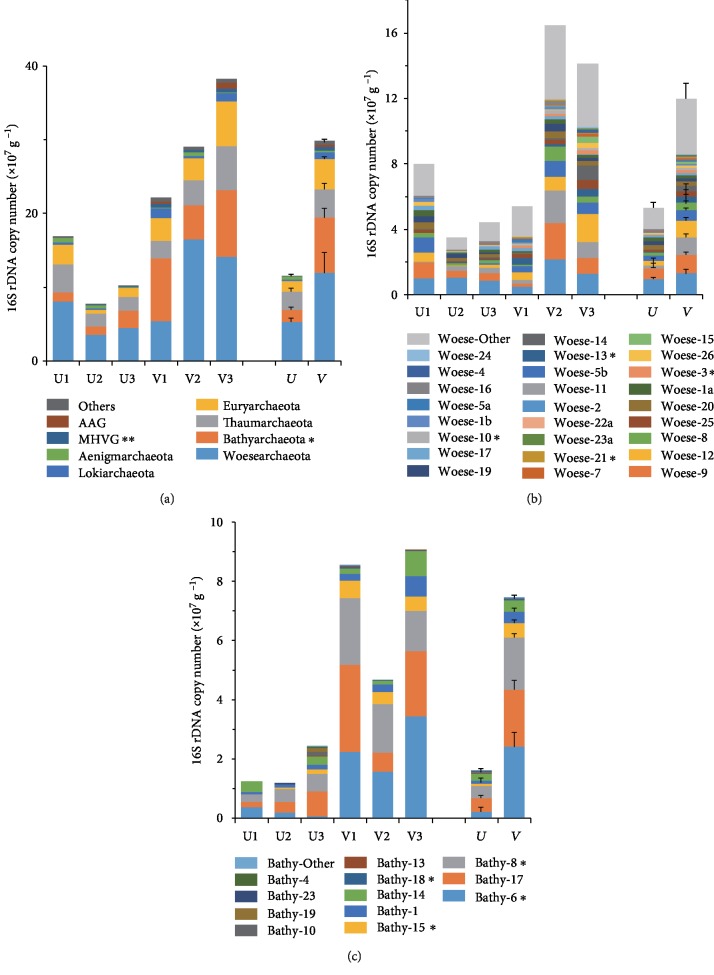
Comparison of archaeal absolute abundances (16S rDNA copy numbers) between vegetated and unvegetated sediments: (a) at the phylum level (MHVG, Marine Hydrothermal Vent Group; AAG, Ancient Archaeal Group; Others, archaeal phyla with relative abundances < 1%); (b) subclades of *Woesearchaeota*; (c) subclades of *Bathyarchaeota*. Those taxa with significant differences between the two niches at the 0.05 level are indicated with ^∗^.

The 16S rRNA gene copy number of each archaeal subgroup in a sample was calculated by multiplying the total archaeal quantity determined by qPCR with its corresponding proportion in that sample obtained by analyzing the high-throughput sequencing dataset [28] (Figure 4, [Supplementary-material supplementary-material-1]). Compared with those in the unvegetated samples ((5.31 ± 1.12) × 10^6^ copies g^−1^ sediment), the absolute quantity of *Woesearchaeota* almost doubled in the vegetated samples ((1.19 ± 0.27) × 10^7^ copies g^−1^ wet sediment; Figure 4(a)). The subclades Woese-3, Woese-10, Woese-13, and Woese-21 exhibited 1.65 ± 0.26, 1.62 ± 0.26, 3.56 ± 0.75, 0.73 ± 0.02 × 10^5^ copies g^−1^ sediment, respectively, and were significantly more abundant in the vegetated sediments (*P* < 0.03, Figure 4(b)). Similarly, the copy number of *Bathyarchaeota* ((7.43 ± 1.13) × 10^6^ copies g^−1^ sediment) was approximately 4 times that in the unvegetated samples (*P* = 0.016), which was apparently due to the higher abundance of four of its subclades, Bathy-6, Bathy-8, Bathy-15, and Bathy-18 (*P* < 0.03) (Figures 4(a), 4(c)). Apart from these major taxa, the minor Marine Hydrothermal Vent Group (MHVG) lineage appeared at 10 times higher abundance ((4.65 ± 0.63) × 10^5^ copies g^−1^ sediment) in vegetated sediments (*P* = 0.006) (Figure 4(a)). The classes Group C3 (vegetated vs. unvegetated, (26.06 ± 4.79) × 10^5^ copies g^−1^ sediment vs. (7.42 ± 1.1) × 10^5^ copies g^−1^ sediment; *P* = 0.036) and *Thermoplasmata* ((34.49 ± 7.28) × 10^5^ copies g^−1^ sediment vs. (7.52 ± 1.83) × 10^5^ copies g^−1^ sediment; *P* = 0.043) were also dramatically stimulated in the vegetated sediments ([Supplementary-material supplementary-material-1]).

### 3.3. Comparison of Archaeal Diversity between Vegetated and Unvegetated Sediments

After normalization, the OTU numbers of the vegetated and unvegetated samples were estimated to be on average 154 and 143, respectively ([Supplementary-material supplementary-material-1]). Values of Shannon, Simpson, and Chao1 diversity indexes ranged from 6.39 to 6.96, 0.98 to 0.99, and 211.68 to 353.84, respectively ([Supplementary-material supplementary-material-1]). No significant differences in OTU richness or the Shannon and Simpson indexes were observed between the seagrass-colonized and the bare sediments (*P* > 0.05); only the Chao1 index appeared to be moderately higher in the seagrass-colonized sediments (*P* = 0.08, Table 1).

## 4. Discussion

### 4.1. *Woesearchaeota* Predominated in the Archaeal Community of the *Z. marina* Seagrass Meadow

Here, we present the archaeal community diversity and distribution patterns in a *Z. marina* seagrass meadow for the first time. High-throughput sequencing results showed that archaeal communities in the *Z. marina* seagrass meadow sediments were highly (more than 40%) represented by *Woesearchaeota*, which was inconsistent with the results obtained in the *Z. noltii* meadow sediments based on clone library [11], in which most archaeal sequences were phylogenetically associated with *Methanobacteria*. The results suggested that different archaeal communities could associate with different seagrass species or depend on the variable local environmental conditions of seagrass meadows. Certainly, sequencing depth and primer bias could cause deviation in the results.

Based on previous reports, *Woesearchaeota* might be involved in anaerobic carbon cycling [29] and presented high proportions in certain highly productive environments, such as 20% in the cyanobacteria-dominated Zhushan Bay [30, 31], 30-60% in Bohai and Yellow Sea surface sediments [32], and approximately 20% in mangroves [16, 33, 34]. It seemed that *Woesearchaeota* presented much higher proportions in the *Z. marina* seagrass system than in mangroves. It is assumed that the source and quality of sediment organic matter regulate the relative abundance of *Woesearchaeota*. The pool of sediment organic matter in seagrass meadows is composed of deposited planktonic or epiphytic algae and seagrass debris as well as root-leaching dissolved organic carbon. The C/N ratios of our seagrass-colonized sediments are around 10 [10], whereas they are generally >20 in mangroves [35, 36], where the sediment organic matter primarily consists of mangrove litter, root exudates, and other terrigenous organic debris. The former is believed to be more conducive to microbial consumption. In addition, latitude could be another factor governing the distribution of *Woesearchaeota* in the *Z. marina* seagrass meadow and mangroves. The *Z. marina* seagrass meadow is located in temperate midlatitudes, while the mangroves are mainly located in tropical low latitudes. Liu et al. [12] noted that most *Woesearchaeota* have been reported in midlatitude environments. Interestingly, the proportions of *Woesearchaeota* obtained in this study were comparable with those in the adjacent Bohai Sea and Yellow Sea surface sediments (30-60%) and much higher than those in the distant East China Sea (approximately 10%) [32], suggesting that the distribution of *Woesearchaeota* might also be driven by geographic distance. Recently, similar geographic segregation was found in the *Woesearchaeota* composition in Chinese lakes from Eastern China to western Xinjiang Province [37].

In total, 24 *Woesearchaeota* subclades were identified in the seagrass system, suggesting high diversity of this phylum in the seagrass meadow. Woese-2 and Woese-9 were the most abundant subclades there. According to sequence origins [12], Woese-2 and Woese-9 were only detected in anoxic environments, and Woese-2 was only observed in saline or hypersaline environments, suggesting that anoxic and saline conditions in the seagrass meadow sediments could contribute to the evolutionary diversity of *Woesearchaeota*.

### 4.2. Selectively Enriched Archaeal Populations in Seagrass-Colonized or Bare Sediments

Though the whole *Woesearchaeota* phylum showed similar relative proportions in seagrass-colonized and bare sediments (Figure 1(a)), its subclades Woese-3 and Woese-21 tended to be more abundant in seagrass-colonized sediments (Figure 1(b)). Woese-3 prefers oxic environments [12], which was selectively enriched in vegetated sediments, possibly due to increased oxygen around the plant rhizosphere [6]. In addition, there were higher Chl-*a* concentrations in the overlying waters of seagrass-colonized sites [10], the higher phytoplankton stock in the water and putatively higher biomass of microphytobenthos might bring more labile organic matters to the sediment surface [38, 39]. This suggested that Woese-3 could adapt to the labile organic substrate supply in the seagrass-colonized environments. There is little available information on the niche preference of Woese-21, and it is currently only known that Woese-21 adapts to broad oxic and salinity conditions [12]. Woese-20 presented an opposite pattern that was selectively enriched in the bare sediments. Most Woese-20 was found in anoxic habitats [12], and thus, seagrass-associated oxygen release could inhibit Woese-20 around the rhizosphere.

As the second most abundant phylum, *Bathyarchaeota* was significantly enriched in seagrass-colonized sediments (vegetated vs. unvegetated, 26.17% vs. 15.44%) (Figure 1(a)), which was consistent with the result for mangroves [13, 16], where *Bathyarchaeota* generally accounted for more than 40% of the relative abundance in archaeal community, and showed significantly higher proportions in mangrove sites than the nearby mud-flat sediments [16]. *Bathyarchaeota* has been reported to contribute importantly to global carbon cycling, considering its ability to assimilate a wide variety of organic compounds, including detrital proteins, acetate, aromatic compounds, and/or other organic substrates [13, 40–43], and it generally dominated in the archaeal community of the marine subsurface sediments combined with a large amount of carbon deposited on the subseafloor [41, 44].

Within *Bathyarchaeota*, the most abundant subclade, Bathy-6, was significantly promoted in the vegetated sediments (Figure 1(c)). Analogously, this subclade accumulated in the sediments dominated by macrophytes [30] and mangroves [16]. The Bathy-6 genome was reconstructed from the suboxic and sulfide-depleted shallow sediment layers, which harbor genes encoding enzymes responsible for degrading extracellular plant-derived mono- and polysaccharides [14, 18]. Seagrass roots release oxygen to sediments, which results in less reducing and sulfide-depleted conditions in seagrass-colonized sediments [6, 45, 46], together with rich seagrass-derived organic matters, and Bathy-6 was well fueled in the sediments. In mangrove wetlands, pH is also an important force shaping the Bathyarchaeotal community structure [16]. The variation in pH of shallow seagrass meadow waters is known to be closely related to photosynthetic activities, which could influence the release of DOC and O_2_ penetration via roots and thus affect the Bathy-6 distribution [10].

In addition to Bathy-6, the abundant Bathy-8 and Bathy-17 subclades showed higher proportions in vegetated sediments. This was in line with the distribution of *Bathyarchaeota* subclades in mangrove sediments [16, 47]. Based on the evidence from enrichment experiments, Bathy-8 can grow using the refractory aromatic polymer lignin as an energy source, during which its relative proportion doubled compared to the initial stage with lignin addition [48]. Furthermore, putative lignin- and aromatic-degrading genes were identified through metagenomic analysis of Bathy-8 [49]. This capacity supports the existence of Bathy-8 in seagrass-colonized sediments containing large amounts of seagrass fibers (57% cellulose, 38% noncellulosic polysaccharides, and 5% lignin) [49, 50]. The metabolic function of Bathy-17 is poorly understood, but according to genomic bins, Bathy-17 can degrade refractory detrital proteins [14]. In addition to plant proteins, many microbial proteins, representing refractory organic matter, were buried in the seagrass sediments [8, 9], and Bathy-17 might contribute to degrading this kind of substrate.

In contrast, *Thaumarchaeota* was selectively enriched in the bare sediments (Figure 1(a)). The identified *Thaumarchaeota* were mainly composed of Group C3, Marine Group I (formerly referred to as Marine Group 1.1a), and Soil Crenarchaeotic Group (formerly Marine Group 1.1b) in this study ([Supplementary-material supplementary-material-1]). The last two classes are important ammonium-oxidizing archaea (AOA) [51], which presented much lower proportions in vegetated sediments that could be linked to lower NH_4_^+^ concentrations in the sediments [10, 52]. The high affinity of seagrass roots for NH_4_^+^ may allow seagrasses to outcompete sediment AOA for NH_4_^+^ [53]. This competitive mechanism explained the low rates of microbial nitrification observed in some seagrass meadows [54–56]. Moreover, the high levels of metals in the vegetated sediments might have strongly influenced the distribution of *Thaumarchaeota* due to the toxicity of metals to AOA, as noted in other studies [12, 57].

### 4.3. Seagrass Colonization Stimulated Archaeal Absolute Abundance

In this study, we applied the integrated high-throughput absolute abundance quantification (iHAAQ) method, which has been demonstrated to evaluate the absolute abundance of bacterial subgroups [28, 58, 59]. A potential bias in our study was that two different sets of archaea-specific primers were applied for high-throughput sequencing (344F/519R) and qPCR (931F/M1100R). In fact, compared with 931F/M1100R, the primer set 344F/519R has slightly different coverages for some major archaeal groups (e.g., *Bathyarchaeota*, *Woesearchaeota*, *Thaumarchaeota*, and *Euryarchaeota*), but contrastingly different coverages for *Korarchaeota*, *Hadesarchaeaeota*, and *Asgardaeota*, as shown by the results of TestPrime 1.0 (https://www.arb-silva.de/search/testprime/) [24]. Nevertheless, none of *Korarchaeota*, *Hadesarchaeaeota*, and *Asgardaeota* occurred in our samples; we therefore believe the bias in absolute abundance due to the primers is minor in our study.

The quantities of *Woesearchaeota* in the vegetated sediments increased to twice those in the bare sediments, and the quantities of Woese-3, Woese-10, Woese-13, and Woese-21 were significantly higher in the vegetated sediments (Figures 4(a) and 4(b)). Recent studies found that *Woesearchaeota* was strongly stimulated in Taihu Lake surface sediments during a cyanobacterial bloom [30] and that *Woesearchaeota* might be involved in the anaerobic carbon cycling [29]. It was also suggested that *Woesearchaeota* might perform symbiotic or pathogenic lifestyles due to missing the core biosynthetic pathways [29]. Considering the significantly higher total archaeal abundance in the vegetated sediments (Figure 3), it is possible that high abundance of other archaeal subgroups supplies more byproducts for *Woesearchaeota* and stimulates their growth and persistence in the vegetated sites [12]. Besides, *Woesearchaeota* was also usually found to be the most abundant in anaerobic nitrogen-removing wastewater treatment sludge [60]. The analysis of the genomics of *Woesearchaeota* indicated that this archaea group harbored nitrogen removal genes such as *nirK* and *nosZ* [12], suggesting that *Woesearchaeota* might participate in nitrogen removal processes and contribute to lower the DIN level in the vegetated sediments.

*Bathyarchaeota* and its subclades Bathy-6, Bathy-8, Bathy-15, and Bathy-18 were strongly stimulated by seagrass colonization (Figure 4(c)). Also, Pan et al. reported that the abundance of *Bathyarchaeota* in the mangrove sediments was significantly higher than that in the mud-flat sediments, and it showed positive correlation with sediment TOC content [16]. There was no difference in TOC content between the seagrass-colonized and bare sediments, but the former should have more bioavailable organic matter with lower TOC : TN, such as root exudates [10], which could promote the growth of *Bathyarchaeota*.

*Thermoplasmata*, a deeply branching class within the phylum *Euryarchaeota*, also exhibited higher absolute quantity in vegetated sediments. According to a genomic analysis, *Thermoplasmata* has the capacity to degrade detrital proteins and long-chain fatty acids [32, 41]. It recurrently coexists in the same sedimentary niches with *Bathyarchaeota* and shares the organic substrates [61].

## 5. Conclusions

In this study, we first analyzed the diversity of archaea in a *Z. marina* seagrass meadow and evaluated the influence of seagrass colonization on archaeal community structures and abundance through high-throughput sequencing and qPCR technologies. In particular, we examined the distribution patterns of subclades of *Woesearchaeota* and *Bathyarchaeota* in the sediments in combination with both their relative proportions and absolute quantities. We found that *Woesearchaeota* dominated (approximately 42%) in archaeal communities of the seagrass system, followed by *Bathyarchaeota* (21%), and the relative proportions of these two phyla were comparable in the two habitats. However, some subclades of the two groups were selectively enriched in vegetated or bare sediments. *Thaumarchaeota* adapted better to the bare sediments, while other phyla presented no heterogeneity in the two niches. The absolute quantity of the total Archaea was significantly stimulated by seagrass colonization, within which of *Bathyarchaeota* in vegetated sediments increased to nearly 5-fold of that in bare sediments. In addition, the subclades Woese-3, Woese-10, Woese-13, and Woese-21 were significantly more abundant in the vegetated sediments. Our studies highlight the niche preferences of archaeal individuals, especially the subclades of the abundant *Woesearchaeota* and *Bathyarchaeota* phyla. The results supply some valuable references for the ecological significance of archaeal lineages in marine sediments.

## Figures and Tables

**Table 1:** Comparison of alpha diversity estimators (mean ± SE) of the whole archaeal community in the vegetated and unvegetated sediments (*n* = 3).

Diversity index	Vegetated	Unvegetated	*P*
OTU richness	154 ± 7.8	143 ± 6.8	0.36
Shannon	6.6 ± 0.16	6.7 ± 0.08	0.86
Simpson	0.98 ± 0.01	0.98 ± 0.01	0.51
Chao1	331 ± 17.5	241 ± 35.7	0.08

## Data Availability

The high-throughput sequencing data was available in the NCBI Sequence Read Archive under accession number PRJNA385281. The data used to support the findings of this study are included within the article and the supplementary information files.

## Supplementary Materials

Supplementary Materials Supplementary Figure S1: The phylogenetic maximum-likelihood (ML) tree was constructed for all Woesearchaeota 16S rRNA gene sequences obtained in the study with bacterial 16S rRNA gene sequences as the outgroup. Sequences for typical Woesearchaeota subclades according to the nomenclature promoted by Liu are used as major references for constructing the phylogenetic tree [12]. The ML tree was built with the “FastTree” program and edited with the online tool iTOL (http://itol.embl.de/). Supplementary Figure S2: The phylogenetic maximum-likelihood (ML) tree was built for all Bathyarchaeota 16S rRNA gene sequences obtained in this study with bacterial 16S rRNA gene sequences as the outgroup. Sequences for typical Bathyarchaeota subclades according to the nomenclature promoted by Zhou [13] are used as major references for constructing the phylogenetic tree. The ML tree was built with the “FastTree” program and edited with the online tool iTOL (http://itol.embl.de/). Supplementary Table S1: Summary of archaeal sequence information and alpha diversity in all sediment samples. Supplementary Table S2: Comparison of the relative proportions and 16S rRNA gene copy numbers of archaeal lineages based on SILVA (v. 128) between the vegetated and unvegetated sediments. The pairwise differences were examined using t-tests (n = 3), and significant differences (P < 0.05) were highlighted in bold.
