# Supplementary material for: Community Structure and Abundance of Archaea in a Zostera marina Meadow: A Comparison between Seagrass-Colonized and Bare Sediment Sites
Source: Archaea. 2019 Nov 18;2019:5108012. doi: 10.1155/2019/5108012 (PMC6885780; doi:10.1155/2019/5108012)
Supplement: Supplementary Materials — Supplementary Figure S1: The phylogenetic maximum-likelihood (ML) tree was constructed for all Woesearchaeota 16S rRNA gene sequences obtained in the study with bacterial 16S rRNA gene sequences as the outgroup. Sequences for typical Woesearchaeota subclades according to the nomenclature promoted by Liu are used as major references for constructing the phylogenetic tree [12]. The ML tree was built with the “FastTree” program and edited with the online tool iTOL (http://itol.embl.de/). Supplementary Figure S2: The phylogenetic maximum-likelihood (ML) tree was built for all Bathyarchaeota 16S rRNA gene sequences obtained in this study with bacterial 16S rRNA gene sequences as the outgroup. Sequences for typical Bathyarchaeota subclades according to the nomenclature promoted by Zhou [13] are used as major references for constructing the phylogenetic tree. The ML tree was built with the “FastTree” program and edited with the online tool iTOL (http://itol.embl.de/). Supplementary Table S1: Summary of archaeal sequence information and alpha diversity in all sediment samples. Supplementary Table S2: Comparison of the relative proportions and 16S rRNA gene copy numbers of archaeal lineages based on SILVA (v. 128) between the vegetated and unvegetated sediments. The pairwise differences were examined using t-tests (n = 3), and significant differences (P < 0.05) were highlighted in bold. [file 5108012.f1.pdf]

Supplementary Material for

Community structure and abundance of archaea in a *Zostera marina* meadow: A comparison between seagrass-colonized and bare sediment sites

Pengfei Zheng [1, 2], Chuantao Wang[3], Xiaoli Zhang [1*], Jun Gong [1, 4, 5*]

[1]CAS Key Laboratory of Coastal Environmental Processes and Ecological Remediation, Yantai Institute of Coastal Zone Research, Chinese Academy of Sciences, Yantai 264003, China.

[2]University of Chinese Academy of Sciences, Beijing 100049, China.

[3]Shandong Oriental Ocean Sci-tech Co., ltd, Yantai 264003, China.

[4]Laboratory of Microbial Ecology and Matter Cycles, School of Marine Sciences, Sun Yat-Sen University, Zhuhai 519082, China.

[5]Southern Marine Science and Engineering Guangdong Laboratory (Zhuhai), Zhuhai, China

Corresponding author

Xiaoli Zhang, Email: xlzhang@yic.ac.cn

Jun Gong, Email: gongj27@mail.sysu.edu.cn

Contents of this file

Figures S1 and S2 Tables S1 and Tables S2

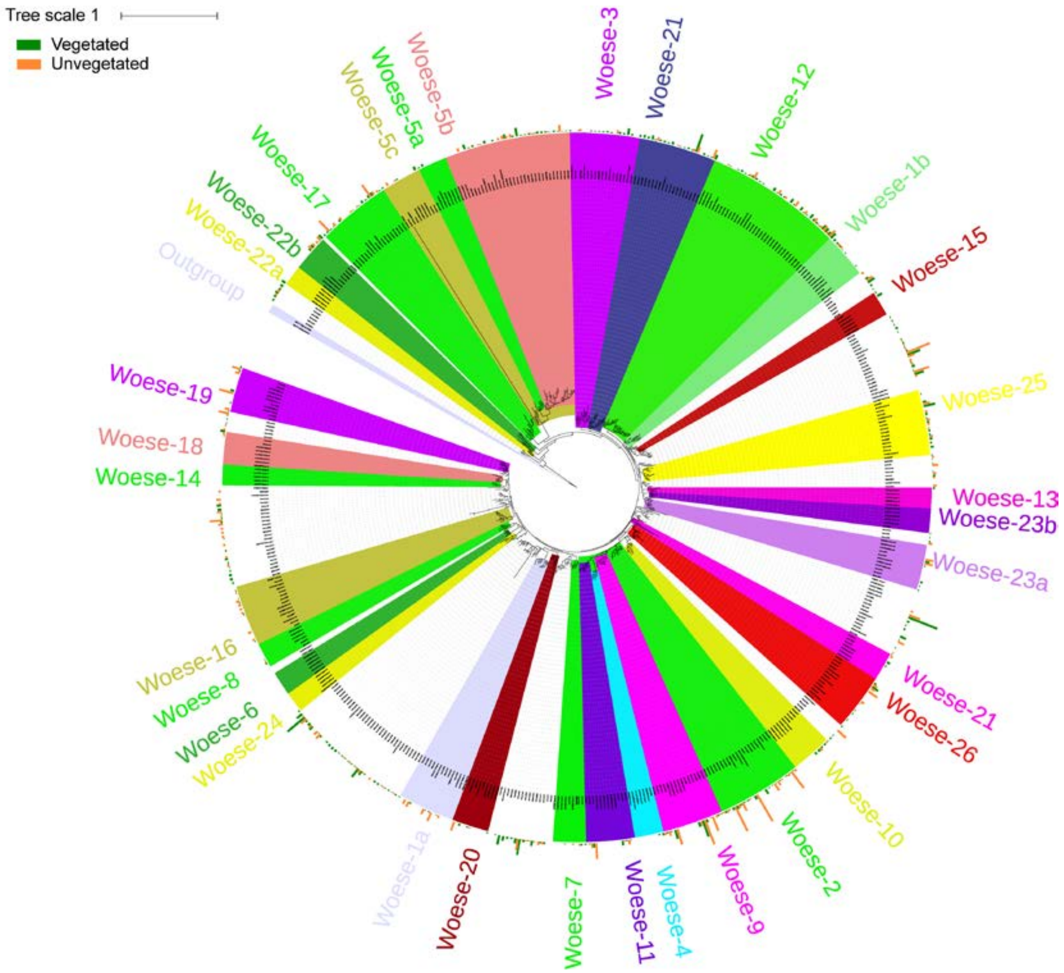

Figure S1 The phylogenetic maximum-likelihood (ML) tree was constructed for all *Woesearchaeota* 16S rRNA gene sequences obtained in the study with bacterial 16S rRNA gene sequences as the outgroup. Sequences for typical *Woesearchaeota* subclades according to the nomenclature promoted by Liu et al. (2018) are used as major references for constructing the phylogenetic tree. The ML tree was built with the "FastTree" program and edited with the online tool iTOL (http://itol.embl.de/)

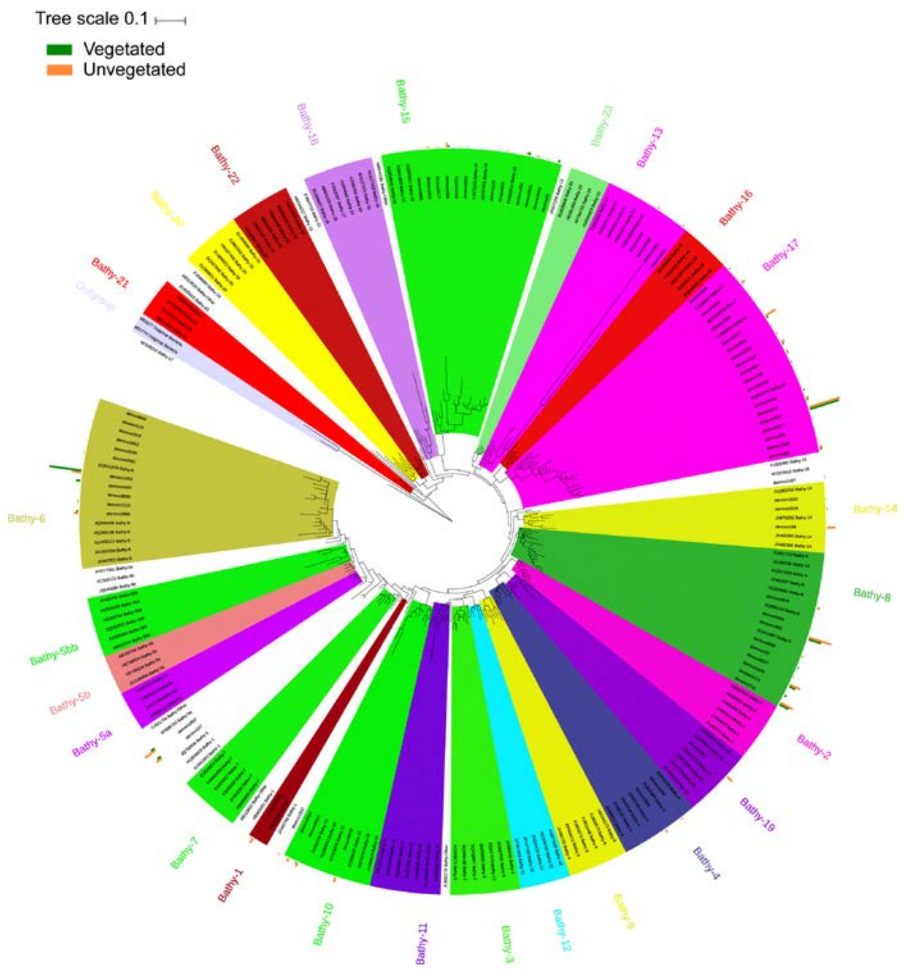

Figure S2 The phylogenetic maximum-likelihood (ML) tree was built for all *Bathyarchaeota* 16S rRNA gene sequences obtained in this study with bacterial 16S rRNA gene sequences as the outgroup. Sequences for typical *Bathyarchaeota* subclades according to the nomenclature promoted by Zhou et al. (2018) are used as major references for constructing the phylogenetic tree. The ML tree was built with the "FastTree" program and edited with the online tool iTOL (http://itol.embl.de/)

Table S1 Summary of archaeal sequence information and alpha diversity in all sediment samples

**Sequence information**					**Alpha diversity**			
Sample ID	Raw seqs	Quality seqs	After discarding chimera seqs	After discarding singleton seqs	OTU Richness	Chao1	Shannon	Simpson
V1	28136	21783	14139	6326	141	296.88	6.39	0.98
V2	22951	10501	9010	8343	167	353.84	6.96	0.99
V3	14610	11770	6839	6326	152	343.40	6.62	0.98
U1	13293	5402	4840	4596	137	198.72	6.74	0.99
U2	7618	3643	3386	3285	135	211.68	6.52	0.98
U3	14028	5843	5305	5046	156	311.70	6.82	0.99

Table S2 Comparison of the relative proportions and 16S rRNA gene copy numbers of archaeal lineages based on SILVA (v. 128) between the vegetated and unvegetated sediments. The pairwise differences were examined using *t*-tests (n=3)[a]

Taxonomy	% Relative proportion (mean ± SE)			Copy numbers (mean ± SE) ($x10^6$ copies g^{-1} sediment)		
	Unvegetated	Vegetated	*P*	Unvegetated	Vegetated	*P*
Woesearchaeota	45.45±0.98	39.34±7.72	0.556	5.31±1.13	11.98±2.75	0.141
Woese-10	0.15±0.12	0.55±0.07	0.076	0.01±0.01	0.16±0.03	**0.014**
Woese-11	2.34±0.81	3.53±1.43	0.586	0.22±0.07	1.08±0.42	0.173
Woese-12	2.03±0.57	3.19±0.58	0.311	0.27±0.12	1.01±0.31	0.139
Woese-13	0.41±0.17	1.26±0.32	0.130	0.04±0.02	0.36±0.07	**0.027**
Woese-14	0.36±0.04	0.99±0.52	0.385	0.04±0.01	0.35±0.21	0.305
Woese-15	0±0	0.35±0.24	0.297	0±0	0.13±0.09	0.315
Woese-16	0.95±0.1	0.28±0.23	0.092	0.10±0.01	0.08±0.07	0.796
Woese-17	1.35±0.21	0.58±0.18	0.090	0.16±0.04	0.15±0.04	0.877
Woese-19	2.15±0.6	0.67±0.37	0.160	0.24±0.07	0.21±0.11	0.834
Woese-1a	1.59±0.32	0.59±0.15	0.082	0.2±0.07	0.18±0.05	0.808
Woese-1b	0.58±0.26	0.35±0.13	0.551	0.08±0.04	0.10±0.03	0.877
Woese-2	9.24±1.87	4.35±1.27	0.153	0.96±0.05	1.31±0.39	0.499
Woese-20	2.26±0.14	0.80±0.30	**0.023**	0.27±0.07	0.25±0.09	0.912
Woese-21	0±0	0.26±0.03	**0.003**	0±0	0.07±0	**0**
Woese-22a	0±0	0.04±0.03	0.374	0±0	0.01±0.01	0.374
Woese-23a	0±0	0.09±0.07	0.374	0±0	0.03±0.03	0.374
Woese-24	0±0	0.09±0.07	0.374	0±0	0.03±0.03	0.374
Woese-25	0.90±0.080	1.05±0.14	0.510	0.11±0.03	0.33±0.09	0.125
Woese-26	0.75±0.30	0.40±0.14	0.435	0.10±0.06	0.13±0.06	0.798
Woese-3	0.11±0.09	0.55±0.03	**0.018**	0.01±0.01	0.17±0.03	**0.011**

Woese-4	0.07±0.06	0.13±0.11	0.709	0.01±0.01	0.05±0.04	0.445
Woese-5a	0.12±0.1	0.31±0.26	0.599	0.02±0.02	0.07±0.06	0.537
Woese-5b	2.11±1.39	2.23±0.46	0.951	0.34±0.24	0.67±0.15	0.395
Woese-7	0.18±0.15	0.30±0.07	0.599	0.02±0.02	0.10±0.03	0.167
Woese-8	1.60±0.06	1.48±0.63	0.889	0.18±0.03	0.45±0.18	0.301
Woese-9	5.36±0.32	3.63±1.70	0.460	0.63±0.15	1.11±0.49	0.482
Woese-Other	10.84±0.51	11.31±1.83	0.851	1.29±0.30	3.40±0.66	0.077
Bathyarchaeota	15.44±3.87	26.17±5.40	0.258	1.61±0.33	7.43±1.13	**0.016**
Bathy-1	1.01±0.28	1.25±0.23	0.609	0.1±0.02	0.39±0.12	0.125
Bathy-10	0.44±0.36	0.04±0.03	0.417	0.04±0.04	0.01±0.01	0.477
Bathy-13	0.07±0.06	0.06±0.03	0.908	0.01±0.01	0.02±0.01	0.430
Bathy-14	1.67±0.70	1.16±0.43	0.635	0.22±0.09	0.39±0.19	0.552
Bathy-15	0.75±0.34	1.77±0.34	0.160	0.07±0.03	0.49±0.04	**0.002**
Bathy-17	4.63±1.69	7.10±2.66	0.557	0.46±0.16	1.93±0.55	0.103
Bathy-18	0±0	0.13±0.03	**0.035**	0±0	0.04±0.01	**0.005**
Bathy-19	0.36±0.30	0±0	0.374	0.04±0.03	0±0	0.374
Bathy-23	0.19±0.15	0±0	0.374	0.01±0.01	0±0	0.374
Bathy-4	0.15±0.12	0±0	0.374	0.01±0.01	0±0	0.374
Bathy-6	1.80±0.47	8.15±1.16	**0.014**	0.21±0.07	2.41±0.45	**0.017**
Bathy-8	4.30±1.16	6.48±1.63	0.423	0.42±0.08	1.75±0.22	**0.010**
Bathy-Other	0.07±0.06	0.02±0.02	0.525	0.01±0.01	0±0	0.751
Thaumarchaeota	21.46±1.35	12.6±1.23	**0.017**	2.51±0.56	3.89±0.88	0.338
Group C3	6.59±0.62	8.67±0.77	0.16	0.74±0.11	2.61±0.48	**0.036**
Marine Benthic Group A	0±0	0.02±0.02	0.374	0±0	0±0	0.374
Marine Group I	8.04±2.16	0.99±0.40	0.059	0.80±0.08	0.33±0.14	0.072
Soil Crenarchaeotic Group (SCG)	6.84±2.73	2.80±0.75	0.309	0.97±0.54	0.92±0.32	0.951
South African Gold Mine Gp 1 (SAGMCG-1)	0±0	0.04±0.04	0.374	0±0	0.02±0.01	0.374
Euryarchaeota	10.81±2.29	13.32±1.33	0.482	1.4±0.52	4.04±0.83	0.093
Halobacteria	0.37±0.15	0.08±0.02	0.192	0.05±0.03	0.02±0.01	0.491
Methanobacteria	3.57±1.67	1.42±0.28	0.358	0.52±0.32	0.45±0.14	0.878
Methanomicrobia	0.44±0.27	0.37±0.14	0.86	0.07±0.05	0.11±0.04	0.639
Thermoplasmata	6.42±1.32	11.45±1.42	0.101	0.75±0.18	3.45±0.73	**0.043**
Aenigmarchaeota	3.73±1.16	0.89±0.37	0.13	0.41±0.12	0.26±0.11	0.476
Deep Sea Euryarchaeotic Group (DSEG)	3.73±1.16	0.89±0.37	0.13	0.41±0.12	0.26±0.11	0.476

Lokiarchaeota	1.86±0.56	3.33±1.24	0.427	0.21±0.07	0.91±0.27	0.108
Marine Hydrothermal Vent Group (MHVG)	0.44±0.19	1.64±0.31	0.055	0.04±0.02	0.47±0.06	**0.006**
Miscellaneous Euryarchaeotic Group (MEG)	0.26±0.13	0.68±0.22	0.255	0.02±0.01	0.18±0.04	**0.03**
Altiarchaeales	0.16±0.09	0.04±0.04	0.387	0.02±0.02	0.02±0.01	0.791
Ancient Archaeal Group (AAG)	0±0	1.19±0.51	0.132	0±0	0.38±0.19	0.188
AK8	0.07±0.06	0.11±0.07	0.73	0.01±0.01	0.03±0.01	0.327
Diapherotrites	0.07±0.06	0.21±0.11	0.41	0.01±0.01	0.06±0.03	0.242
WSA2	0.24±0.20	0.16±0.08	0.772	0.04±0.03	0.05±0.02	0.902
20a-9	0.24±0.20	0.16±0.08	0.772	0.04±0.03	0.05±0.02	0.902
Unclassified Archaea	0±0	0.33±0.13	0.117	0±0	0.1±0.04	0.135

[a] Significant differences ($P < 0.05$) are highlighted in bold.
